# Exploring Glypican-3 targeted CAR-NK treatment and potential therapy resistance in hepatocellular carcinoma

**DOI:** 10.1371/journal.pone.0317401

**Published:** 2025-01-22

**Authors:** Lei Yang, Kien Pham, Yibo Xi, Qunfeng Wu, Dongfang Liu, Keith D. Robertson, Chen Liu

**Affiliations:** 1 Department of Pathology, Yale School of Medicine, Yale University, New Haven, Connecticut, United States of America; 2 Department of Pathology and Laboratory Medicine, New Jersey Medical School, Rutgers University, Newark, New Jersey, United States of America; 3 Department of Molecular Pharmacology and Experimental Therapeutics, Mayo Clinic, Rochester, Minnesota, United States of America; Center for Research and Technology Transfer, VIET NAM

## Abstract

Hepatocellular carcinoma (HCC) is the most prevalent form of primary liver cancer and the second leading cause of cancer-related mortality globally. Despite advancements in current HCC treatment, it remains a malignancy with poor prognosis. Therefore, developing novel treatment options for patients with HCC is urgently needed. Chimeric antigen receptor (CAR)-modified natural killer (NK) cells have demonstrated potent anti-tumor effects, making them as a promising immunotherapy strategy for cancer treatment. Glypican-3 (GPC3), a cell surface oncofetal glycoprotein, is highly expressed in most HCC tissues, but not in normal tissues, and functions as a key driver of carcinogenesis. Given its high expression level on the cell surface, GPC3 is considered as an attractive immunotherapy target for HCC. In this study, two GPC3-specific CAR-NK cells, NK92MI/HN3 and NK92MI/HS20, were established using NK92MI cells, a modified IL-2-independent NK cell line. These cell lines were engineered with third generation GPC3-specific CARs, and their activities were subsequently evaluated in the treatment of HCC. We found that NK92MI/HN3 cells, rather than NK92MI/HS20 cells, exhibited a significant cytotoxicity effect against GPC3^+^ HepG2 cells *in vitro* and efficiently suppressed tumor growth in a xenograft model using NSG mice. In addition, irradiated NK92MI/HN3 cells displayed similar anti-tumor efficacy to unirradiated NK92MI/HN3 cells. Furthermore, we observed that NK92MI/HN3 cells showed higher killing activity against the GPC3 isoform 2 overexpression cell line (Sk-Hep1-v2) than those with GPC3 isoform 1 overexpression cell line (Sk-Hep1-v1). This suggest that the presence of different GPC3 isoforms in HCC may impact the cytotoxicity activity of NK92MI/HN3 cells and potentially influence therapeutic outcomes. These findings highlight the effective anti-HCC effects of NK92MI/HN3 cells and reveal the role of GPC3 isoforms in influencing therapy outcomes, suggesting that isoform analysis should be considered to optimize CAR-NK therapies to improve patient outcomes.

## 1. Introduction

Liver cancer ranks as the sixth most commonly diagnosed cancer and the fourth leading cause of cancer-related death worldwide, with continuously increasing incidence and mortality rates, in recent years [1]. By 2030, the burden of liver cancer is projected to exceed 1 million cases [2]. Hepatocellular carcinoma (HCC), accounting for 75%-85% of all diagnosed cases, is the most common type of primary liver cancer [1]. Despite extensive exploration of therapeutic options, HCC remains poor prognosis due to postoperative recurrence and metastasis. Therefore, developing novel strategies and long-life therapies for the patients with HCC are still urgently needed.

Natural killer (NK) cells mediate potent cytotoxicity against tumor cells, making them attractive candidates for effective immunotherapies in the treatment of HCC [3,4]. NK cells possess the unique ability to recognize target cells via a major histocompatibility complex (MHC)-independent mechanism, allowing them to directly eliminate tumor cells, without being sensitized. Like T cells, NK cells can be genetically modified with chimeric antigen receptors (CARs) that can recognize antigens expressed by tumors. These CAR-engineered NK cells are also equipped with signaling components that enhance NK cell activity, thereby increasing their cytotoxicity against tumor cells. In comparison to CAR-T cell-based immunotherapy, which is known to have toxic side effects, the use of CAR-NK cells offers a promising approach to enhance efficacy while mitigating adverse effects such as acute cytokine release syndrome (CRS), neurotoxicity and graft-versus-host disease (GvHD) [5,6]. Several studies have demonstrated that CAR engineering of NK cells significantly enhances their cytotoxicity against various types of cancers [7–11]. For instance, NK92 cells engineered with CARs targeting CD19 displayed an increased cytotoxicity activity against B-cell malignancies [12]. Similarly, CAR-NK cells designed to recognize CD20 and Flt3 have demonstrated effective anti-tumor effects against B-cell tumors [13]. Thus, CAR-NK cell therapy has emerged as a promising immunotherapy strategy for the treatment of cancers.

Glypican-3 (GPC3), a member of the heparan sulfate proteoglycan family, functions as an oncofetal glycoprotein that is attached to the cell membrane via glycosylphosphatidylinositol (GPI) [14]. Several studies have consistently shown that both mRNA and protein levels of GPC3 are significantly elevated in HCC tissue, but not in healthy adult liver tissues [15]. This overexpression of GPC3 has been found to be strongly correlated with a poorer prognosis in individuals diagnosed with HCC [16,17]. Survival analysis of HCC patients with high GPC3 expression has revealed significantly reduced overall survival compared to those with low GPC3 expression. Moreover, the risk of recurrence after liver resection was found to be increased up to three-fold in HCC patients with high GPC3 expression, as compared to those with low GPC3 expression [18]. Given the oncogene functions and high expression level of GPC3 on the cell surface in HCC, it is considered an attractive target for HCC therapy [19–27].

The human GPC3 gene is transcribed and alternatively spliced into four distinct mRNA isoforms [28], with isoform 2 being the most commonly expressed [29]. All GPC3 isoforms share the same C-terminal subunit, while the N-terminal subunits exhibit slight differences. Indeed, the alternative splicing of variants leading to the generation of different isoforms has been implicated in resistance to immunotherapy, as observed in B-cell acute lymphoblastic leukemia (B-ALL) patients with different CD19 isoforms contributing to CAR-T cell escape and resistance to CAR-T immunotherapy [30]. Isoforms arise from the combination of different exons through RNA alternative splicing, resulting in proteins with diverse biological properties [31]. The differential isoform expression can alter cellular activities, including cell proliferation, drug responsiveness and therapy outcomes [32,33]. The existence of various GPC3 isoforms and their potential impact on CAR-NK cytotoxicity may raise questions about the correlation between alternative mechanisms of GPC3 isoforms and their effects on CAR-NK immunotherapy. Further investigation of the expression and functional properties of GPC3 isoforms in the context of CAR-NK immunotherapy may provide valuable insights into their role in determining the outcomes of immunotherapeutic approaches targeting GPC3 and other antigens with alternative splicing isoforms, and potentially guide the development of more effective CAR-NK therapies for cancer treatment.

In the study, we developed two types of CAR-NK cells, NK92MI/HN3 and NK92MI/HS20, by genetically engineering NK92MI cells, a highly cytotoxic NK cell lines. We then evaluated their function and effectiveness against different HCC cells to identify a promising cell-based therapeutic strategy for HCC and investigate the potential impact of GPC3 isoforms on therapy efficacy.

## 2. Materials and methods

### 2.1 Ethics statement

All experimental manipulation of mice was undertaken following the National Institute of Health Guide for the Care and Use of Laboratory Animals. The protocol was approved by the Committee on the Ethics of Yale University (Protocol Number: 2023–20315).

### 2.2 Cell culture

Human HCC cell lines, including HepG2, Huh7, Huh7.5 and Sk-Hep1, as well as the 293T cell line, were purchased from ATCC (Manassas, VA). HCO2 and LH86 hepatoma cell lines were established in our laboratory [34,35]. 293T cells and HCC cell line HCO2, HepG2, Huh7, Huh7.5, Sk-Hep1, LH86, Sk-Hep1-v1, and Sk-Hep1-v2 cells were maintained in Dulbecco’s minimal essential medium (DMEM) supplemented with 10% (v/v) FBS and antibiotics (100 IU/ml of penicillin and streptomycin) at 37°C in a 5% CO2 air-humidified incubator. Cells were routinely passaged with 0.25% trypsin. Human NK92MI, NK92MI/HN3 and NK92MI/HS20 cells were cultured in α-minimum essential medium supplemented with 0.2 mM inositol, 0.1mM 2-mercaptoethanol, 0.02 mM folic acid, 12.5% horse serum and 12.5% FBS.

### 2.3 Construction of retroviral vector and transduction of NK92MI cells

**Fig 1:**
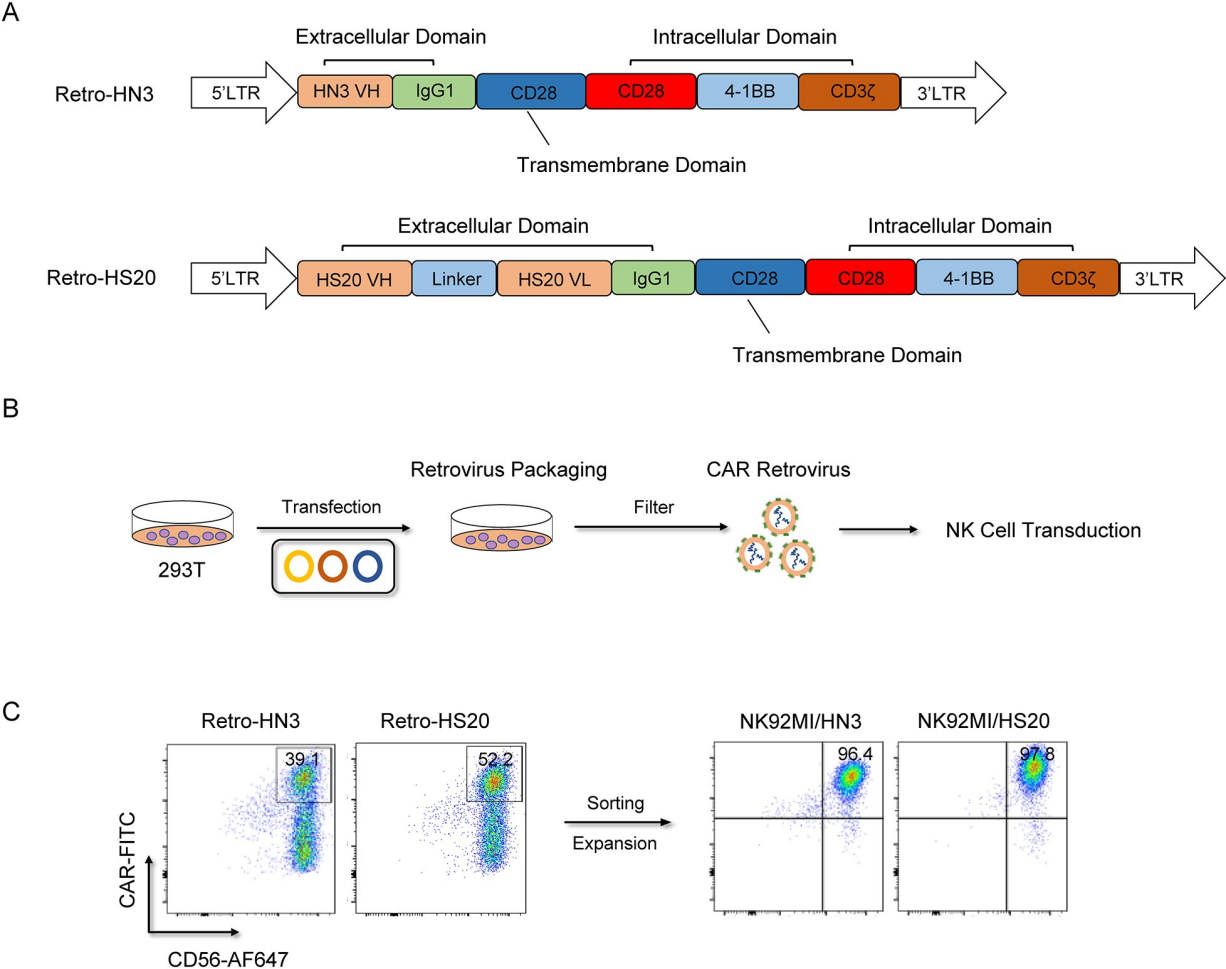
Generation of NK92MI/HN3 and NK92MI/HS20 cells. (A) Schematic design of HN3-CAR and HS20-CAR in SFG retroviral vector. The SFG retroviral vector contains the HN3 or HS20 single chain antibody fragment, a human IgG1 CH2CH3 hinge region and CD28 transmembrane region, followed by the intracellular domains of co-stimulatory CD28, 4-1BB, and the intracellular domain of CD3ζ. (B) Generation of NK92MI/HN3 and NK92MI/HS20 cells. 293T cells were transfected with Retro-HN3 or Retro-HS20 for 72 h for CAR retrovirus packaging and transduced into NK92MI cells. (C) Determination of CAR expression by flow cytometry. NK92MI/HN3 and NK92MI/HS20 cells were harvested after 4–5 days and then stained with anti-CD56 and CAR F(ab)2 domain [IgG (H+L)] for flow cytometry.

Both HN3 and HS20 antibodies, which target GPC3, have demonstrated significant tumor growth inhibition in HCC *in vitro* and *in vivo* [36–38]. The CAR construct used in this study contains the HN3 single-domain antibody or HS20 single-chain antibody fragment, human IgG1 CH2CH3 hinge region, and CD28 transmembrane region, followed by the intracellular domains of co-stimulatory CD28, 4-1BB, and CD3ζ (**Fig 1A**) [39]. For the production of the GPC3-CAR retrovirus, 293T cells were transfected with a combination of plasmids containing the GPC3-CAR in SFG backbone, RDF, and PegPam3, as previously described [39]. After 72 hours, the supernatant containing retroviral particles, Retro-HN3 or Retro-HS20, was harvested. NK92MI cells were then transduced with Retro-HN3 or Retro-HS20 retrovirus using RetroNectin (Clontech) coated plates. Two days post-transduction, the cells were transferred to flask for further expansion. To confirm the surface expression of the CARs, the transduced NK92MI/HN3 and NK92MI/HS20 cells were harvested 4–5 days after transduction. The cells were stained with anti-CD56 (clone HCD56) and CAR F(ab)_2_ domain [IgG (H+L)] (Jackson ImmunoResearch Laboratories INC, PA) for flow cytometry analysis. After validation, the transduced NK92MI/HN3 and NK92MI/HS20 cells were collected, sorted for CAR-positive populations, and expanded for subsequent experiments.

### 2.4 Analysis of NK92MI and CAR-NK92MI cell phenotype

The NK92MI, NK92MI/HN3 and NK92MI/HS20 cells were collected and stained with the following antibodies for flow cytometry: anti-F(ab)_2_ domain [IgG (H+L)] (Jackson ImmunoResearch Laboratories INC, PA), PE-conjugated anti-NKG2C Ab (clone S19005E), AF647-conjugated anti-CD56 Ab (clone HCD56), PECy7-conjugated anti-NKG2A (clone S19004C), FITC-conjugated NKp46 (clone 9E2), PE-conjugated CD94 Ab (clone DX22), APC-conjugated CD69 Ab (clone H1.2F3), APC-Cy7 conjugated NKp44 Ab (clone P44-8), FITC-conjugated CD25 Ab (clone BC96), PE-conjugated NKG2D Ab (clone 1D11), APC-conjugated NKp30 Ab (clone P30-15) and APC-Cy7 conjugated PD-1 Ab (29F.1A12).

### 2.5 Polymerase Chain Reaction (PCR)

The cultured cells were collected for RNA extraction and reverse transcription. cDNA was synthesized from the extracted RNA, and this cDNA was then used as a template for PCR amplification of different GPC3 using the following primers: GPC3F1: 5’-CCGTATAGGGCTAGACTTACAG-3’; GPC3R1: 5’-CAGCTCATGGAGATTGAACTGG-3’; GPC3F2: 5’-TAGAAACTCCCGTGCCAG-3’; GPC3R2: 5’-GCCGTAGAGAGACACATCTGTG-3’. Primers were synthesized by Yale Keck oligo synthesis. The protocol for PCR cycle was as follows: denaturation at 94°C for 30 seconds, annealing at 56°C for 30 seconds, and primer extension at 72°C for 1 minute, for a total of 30 cycles. A final extension step of 10 minutes at 72°C was performed. PCR products were analyzed on a 2% agarose gel stained with SYBR safe and visualized under UV light.

### 2.6 Construction of GPC3 isoform overexpression lentiviral vector

RNA was extracted from HepG2 cells using RNeasy Mini Kit (QIAGEN, MD) according to the manufacture’s instructions, followed by reverse transcription with a cDNA synthesis kit (Thermo Scientific, IL). HepG2 cDNA was used as the template for PCR amplification. The GPC3 isoform 1 and 2 fragments were amplified by PCR with primers as follows: GPC3-V1-F1: 5’-TATATTATATTATATTTATGCTAGCGCCACCATGGCCGGGACCGTGCGCACCGCGT-3’; GPC3-V1-R1: 5’-CTGTAAGTCTAGCCCT-3’; GPC3-V1-F2: 5’-CTTCCTGTGTATAGGGCTAGACTTACAG-3’; GPC3-V1-R2: 5’-ACTAGCGCATATGGATCCTCAGTGCACCAGGAAGAAGAAGCA-3’ and with primers GPC3-V2-F: 5’-TATATTATATTATATTTATGCTAGCGCCACCATGGCCGGGACCGTGCGCACCGCGT-3’ and GPC3-V2-R: 5’-ACTAGCGCATATGGATCCTCAGTGCACCAGGAAGAAGAAGCA-3’, respectively. The pLenti-GIII lentiviral vector, containing puromycin antibiotic selection and green fluorescence protein (GFP) reporter, was used for construction. The obtained GPC3 variant fragments were cloned into the pLenti-GIII plasmid, which contains the NheI and BamHI restriction enzyme sites. The recombinant pLenti-GIII-hGPC3-v1 and pLenti-GIII-hGPC3-v2 plasmids were used for further recombinant lentivirus particle generation.

### 2.7 Lentivirus production and transduction of Sk-Hep1 cells

The 3^rd^ generation packaging system from abm company (Applied Biological Materials Inc, Canada) was utilized for recombinant lentivirus generation. A total of 2 x 10^6^ 293T cells were seeded into a 100 mm dish and incubated overnight. Plasmid transfection was performed using TransIT Lenti reagent (Mirus, WI) according to the manufacturer’s instructions. The 293T cells were transfected with the vector plasmid pLenti-GIII-hGPC3-v1 or pLenti-GIII-hGPC3-v2, along with a helper plasmid mixture, and maintained at 37°C in a 5% CO2 for 48 h. The recombinant lentiviruses were then collected from the supernatant, concentrated, and stored at -80°C for future use. Subsequently, Sk-Hep1 cells were incubated with medium containing GPC3-v1 or GPC3-v2 lentivirus to establish the Sk-Hep-1 GPC3 isoform 1 (Sk-Hep1-v1) or Sk-Hep-1 GPC3 isoform 2 (Sk-Hep1-v2) cell models, respectively. After 48 hours of incubation, the medium was aspirated and replaced with fresh medium containing puromycin at 4 μg/ml for cell selection of Sk-Hep1-v1 and Sk-Hep1-v2. The selective medium containing puromycin was replaced every 2–3 days for up to 2 weeks. Expression validation was subsequently performed.

### 2.8 RNA isolation and qRT-PCR analysis

Total RNA isolation was performed on cultured cells using RNeasy Mini Kit (QIAGEN, MD), followed by reverse transcription with a cDNA synthesis kit (Thermo Scientific, IL). Quantitative real-time polymerase chain reaction (qRT-PCR) was then performed using a two-step SYBR green qPCR assay and the target genes were amplified using following primers. Human *GPC3*, forward: 5’-CATTGGAGGCTCTGGTGATGGA-3’; reverse: 5’-TTGTCCTTCGGAGTTGCCTGCT-3’; Human *GAPDH*, forward: 5’-GTCTCCTCTGACTTCAACAGCG-3’; reverse: 5’-ACCACCCTGTTGCTGTAGCCAA-3’. The qRT-PCR data were acquired using the Step One real-time PCR system (Applied Biosystem, CA). The cycling procedure was as follows: one cycle at 95°C for 30 seconds, followed by 40 cycles at 95°C for 5 seconds and 64°C for 31 seconds. Each assay plate included negative control with no template. The mRNA levels of the gene of interest were normalized to the mRNA levels of GAPDH and analyzed using the 2^−ΔΔCt^ method.

### 2.9 Western blot analysis

Samples from the cultured cells were lysed in ice-cold RIPA buffer (Sigma, MA) containing phosphatase/protease inhibitors (Thermo Scientific, IL). The lysates were then centrifuged at 11,000 g for 10 min at 4°C, and the supernatant was collected and quantified using a BCA protein assay kit (Thermo Scientific, IL). After quantification, cell lysates were loaded and separated by electrophoresis with a 12% sodium dodecyl sulfate-polyacrylamide gel. The protein was then transferred onto a polyvinylidene fluoride membranes (Millipore, MA), which were blocked for 1 h at room temperature in PBS and probed with anti-human GPC3 and GAPDH monoclonal antibodies at 4°C overnight. After washing with PBS containing 0.05% Tween 20 three times at 10-min intervals, the membrane was incubated with goat anti-rabbit IgG or goat anti-mouse IgG secondary antibodies for 1 h at room temperature. After further wash steps, the membranes were treated with the substrate (Thermo Scientific, IL) to visualize the protein bands using the ChemiDoc Imaging System (BioRad, CA).

### 2.10 Cytotoxicity effects of CAR-NK92MI cells to HCC cells

HepG2 cells at a density of 2 x 10^5^ were co-cultured with NK92MI, NK92MI/HN3 and NK92MI/HS20 at E:T ratios of 2.5:1, 5:1 and 10:1 for 24 h and 48 h *in vitro*. GFP florescence signals were observed under a microscope, which served as an indicator to evaluate the killing efficacy of CAR-NK92MI cells. Similarly, Sk-Hep1, Sk-Hep1-v1 and Sk-Hep1-v2 cells at a density of 2 x 10^5^ were cultured in a 24-well plate overnight. NK92MI/HN3 cells were added and incubated with the target cells with an E:T ratio of 5:1 for 24 h and 48 h *in vitro*. Florescence signals were observed under a microscope.

### 2.11 Functional assay of CAR-NK92MI cells

CAR-NK92MI cells were co-cultured with target cells at an E:T ratio of 1:1 for 24h. After incubation, the supernatant from the co-culture system was collected and the production of IFN-γ was detected using an enzyme-linked immunosorbent assay kit (R&D Systems, USA) according to the manufacturer’s instructions. Similarly, the CAR-NK92MI cells were co-cultured with target cells at an E:T ratio of 1:1 for 24h in 96-well V bottom plates. The cells were collected and washed with PBS, and then stained with anti-CD107 for flow cytometry analysis.

### 2.12 Xenogeneic tumor-grafted mouse models

Six to eight-week-old NOD-scid IL2Rgnull (NSG) mice were purchased from the Jackson Laboratory. The NSG mice were randomly divided into four groups. A total of HepG2 cells (1 x 10^6^) were suspended in 50% Matrigel and subcutaneously injected into the right flack of each mouse. When the tumor size reached approximately 100mm^3^, the mice were intravenously (i.v.) injected with NK92MI, NK92MI/HN3, NK92MI/HS20 cells or PBS twice at seven-day intervals. Tumor sizes were monitored and calculated every two days thereafter using the formula V = length x width^2^/2.

The animals will be euthanized using CO_2_ asphyxiation if the tumor size reaches 1.5 cm in diameter or if tumor ulceration occurs. The tumor volume will not exceed 1 cm^3^. In addition to standard endpoint parameters, animals will also be monitored for other clinical signs, including, but not limited to, constipation, weight loss and hunching. Animals showing signs of significant suffering were euthanized before the study’s endpoint to prevent unnecessary pain.

### 2.13 Effects of irradiation on cytotoxicity of NK92MI/HN3 cells *in vitro*

NK92MI/HN3 were irradiated with 5 Gy using an X-ray irradiator. HepG2 cells were co-cultured with irradiated and unirradiated NK cells at various ratios of E:T at 1:1, 2.5:1, 5:1 and 10:1. Florescence signals were observed under the microscope to assess the cytotoxicity of CAR-NK92MI cells towards HepG2 cells.

### 2.14 CFSE cell proliferation assay

1 x 10^6^ irradiated and unirradiated NK92MI/HN3 cells were labeled with CFSE at a final working concentration of 10 μM, according to manufacturer’s instructions (CellTrace^TM^ CFSE Cell Proliferation Kit, Thermo Fisher), and incubated for 20 min at 37°C. The staining was washed using 5 volumes of culture medium. The stained cells were maintained at 37°C in a 5% CO2 for 120 h to analyze the proliferation.

### 2.15 Effects of irradiation on cytotoxicity of NK92MI/HN3 cells *in vivo*

Six-week-old mice were randomly divided into three groups. A total of 1 x 10^5^ HepG2 cells were suspended in 50% Matrigel and subcutaneously injected into the right flack of each mouse. When tumor size reached approximately 100mm^3^, the mice were intravenously (i.v.) injected with irradiated or unirradiated NK92MI/HN3 cells, or PBS, at the indicated time points. Tumor sizes were then monitored and calculated every two days.

### 2.16 Statistics analysis

Data are shown as mean ± SD. All calculations and statistical analyses were performed using Graphpad PRISM 5.0 (GraphPad Software, San Diego, CA) for Mac. Comparisons between groups were conducted using analyses of unpaired t-tests, and P<0.05 was considered as statistically significant.

## 3. Results

### 3.1 Generation of NK92MI/HN3 and NK92MI/HS20 cells

Given its oncogenic function, GPC3 has already been suggested as a potential target for cancer immunotherapy in the treatment of HCC, including CAR-T and CAR-NK strategies [40]. These immunotherapeutic approaches aim to specifically target and eliminate GPC3-expressing tumor cells while minimizing off-tumor effects. Considering the splendid advantages of easy expansion, cultivation and activation, NK92MI cell line with indefinite expansion capacity have been used in clinical practice [41]. To develop an NK cell-based immunotherapy for HCC patients, we genetically engineered NK92MI cells with the third-generation CAR molecules, HN3 or HS20, which specifically target GPC3, a highly expressed antigen in HCC. The human single-domain antibody, HN3, recognized a conformational epitope that requires both the amino and carboxy terminal domains of GPC3 [37], and the HS20 recognizes the heparan sulfate chains on GPC3 [36]. As shown in Fig 1A, we cloned the HN3 single-domain antibody or HS20 single-chain antibody fragment into an SFG retroviral vector. The CAR construct contains human IgG1 hinge CH2-CH3 domain, CD28 transmembrane (TM) domain and intracellular domain, 4-1BB ligand intracellular domain, and CD3zeta intracellular domain. After construction, the 293T cells were transfected with a combination of plasmids containing HN3-CAR or HS20-CAR in the SFG backbone, RDF, and PegPam3, as previously described [42]. The SFG retrovirus particles in the supernatant were utilized to transduce NK92MI cells with the GPC3-specific CAR construct to generate NK92MI/HN3 or NK92MI/HS20 cells (**Fig 1B**). After 4–5 days, the cells were collected and labeled with CD56 and human IgG (H+L) for sorting to enrich the CAR-NK92MI cell population. Flow cytometry analysis demonstrated that more than 96% of CD56+CAR+ NK92MI cells were observed (**Fig 1C**).

### 3.2 Phenotypic characterization of NK92MI/HN3 and NK92MI/HS20 cells

**Fig 2:**
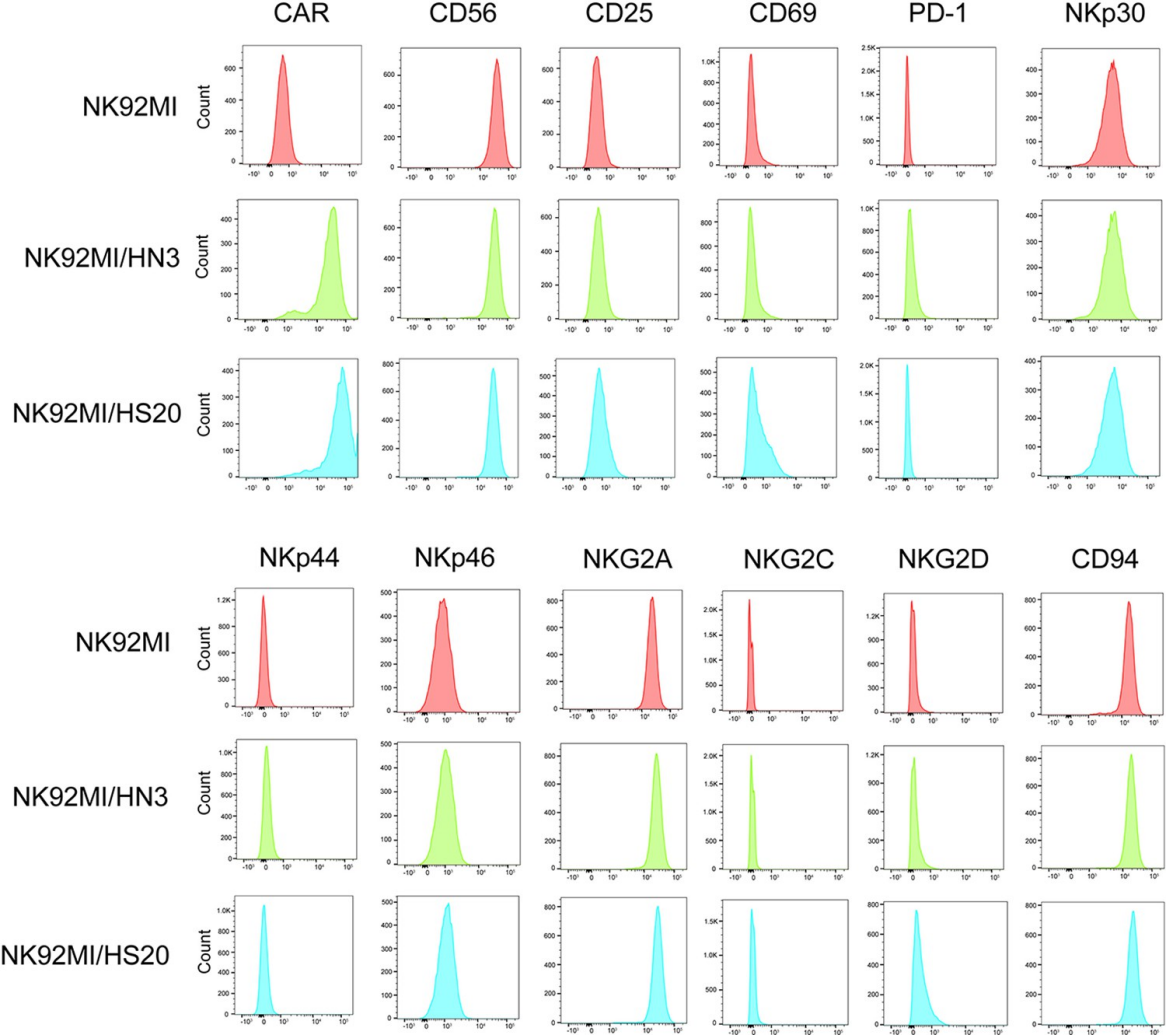
Phenotypic characterization of NK92MI/HN3 and NK92MI/HS20 cells. Representative histograms of the expression of CD56, CD25, CD69, PD-1, NKp30, NKp44, NKp46, NKG2A, NKG2C, NKG2D and CD94 on the cell surface of NK92MI, NK92MI/HN3 and NK92MI/HS20 cells. The expression of these receptors was determined by flow cytometry.

To assess the immunophenotyping of NK92MI, NK92MI/HN3 and NK92MI/HS20 cells, flow cytometry was performed to characterize the expression of several important activating and inhibitory receptors and markers on the surface of the cells, including CD56, CD25, CD69, PD-1, NKp30, NKp44, NKp46, NKG2A, NKG2C, NKG2D and CD94 (**Fig 2**). The results revealed that the expression profile of these receptors and markers did not show significant differences between NK92MI, NK92MI/HN3 and NK92MI/HS20 cells.

### 3.3 Cytotoxicity activity of NK92MI/HN3 and NK92MI/HS20 cells against HepG2 cells *in vitro* and *in vivo*

**Fig 3:**
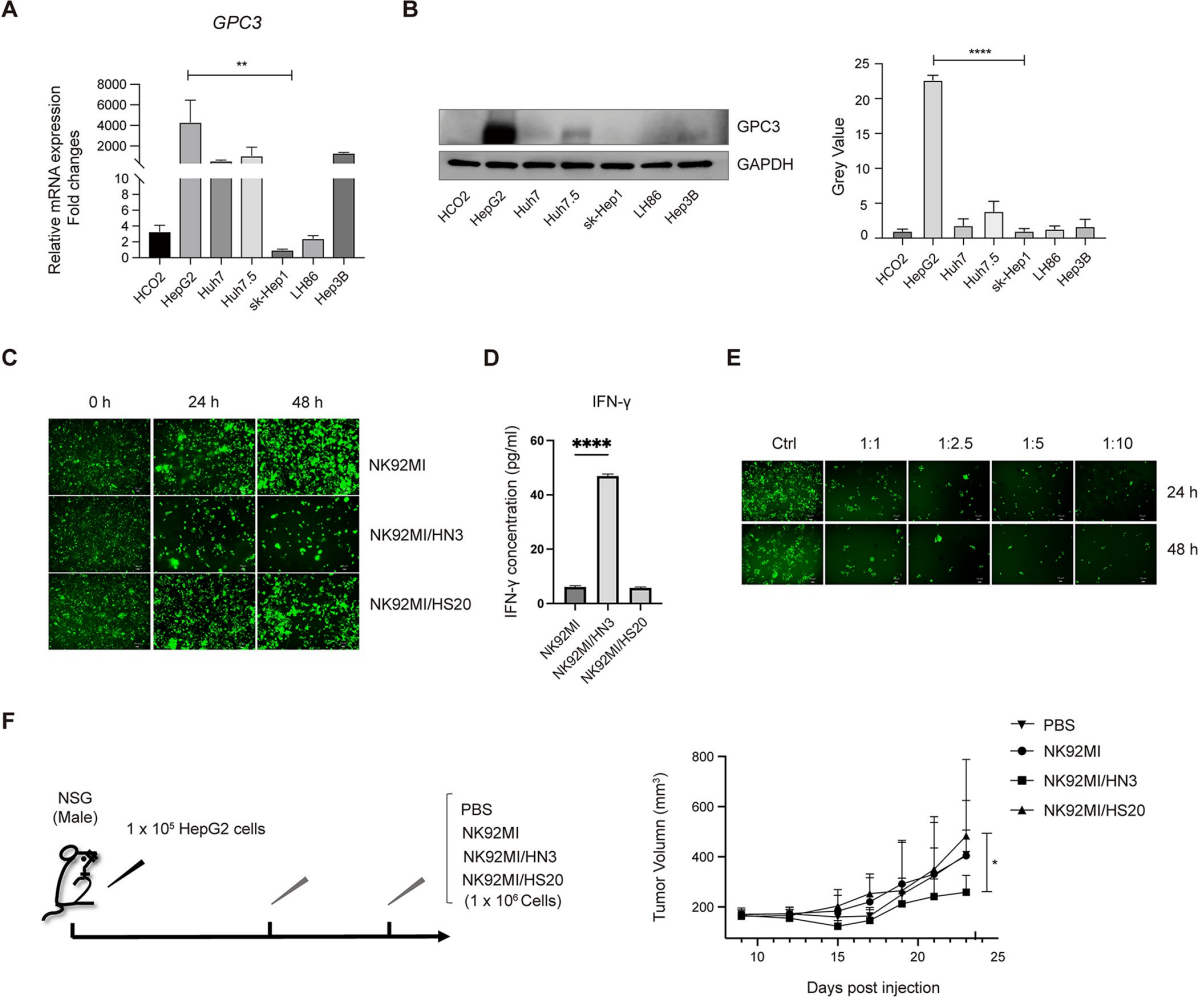
Cytotoxicity activity of NK92MI/HN3 and NK92MI/HS20 cells against HepG2 cells *in vitro* and *in vivo*. (A) mRNA level and (B) protein expression levels of GPC3 in different HCC cell lines. (C) Cytotoxicity activity of NK92MI, NK92MI/HN3 or NK92MI/HS20 against HepG2 cells *in vitro*. GFP signal was observed under the florescence microscope. (D) The IFN-γ production of the co-cultured NK cells with HepG2 cells *in vitro*. IFN-γ concentration was measured in the supernatant. (E) Cytotoxicity activity of NK92MI/HN3 against HepG2 cells at an E:T ratio of 1:1, 2.5:1, 5:1 and 10:1 for different time points. (F) Procedure and growth curve of HepG2 Xenografts treated with NK92MI, or NK92MI/HN3 or NK92MI/HS20 cells. The mRNA levels were normalized with the mRNA levels of *GAPDH*. n = 5 mice per group. Data are represented as mean ± SD. *, P < 0.05; **, P< 0.01; ***, P< 0.001.

To determine the recognition of CAR-NK cells to the GPC3+ HCC cells, we first evaluated the expression of GPC3 in several HCC cell lines, including HCO2, HepG2, Huh7, Huh7.5, Sk-Hep1, LH86 and Hep3B cell lines. As shown in **Fig 3A**, we observed higher mRNA levels of *GPC3* in HepG2, Huh7, Huh7.5 and Hep3B cells, and lower levels in HCO2 and LH86 cells. On the other hand, the protein expression of GPC3 was significantly higher in HepG2 cells (**Fig 3B**), while Huh7, Huh7.5, and Hep3B cells showed relatively lower GPC3 expression levels. Consistent with previous data from other groups, Sk-Hep1 cells did not exhibit detectable GPC3 protein expression [43]. Thus, HepG2 cells were chosen as the cell model for subsequent studies.

To assess the anti-tumor efficacy of different CAR-NK92MI cells, we co-cultured the HepG2 cells with NK92MI, NK92MI/HN3 and NK92MI/HS20 cells and observed the florescence signal under a fluorescence microscope. Compared to the NK92MI and NK92MI/HS20 cells, co-culturing with NK92MI/HN3 cells displayed less GFP fluorescence signals, indicating a high anti-tumor effect against HepG2 cells (**Fig 3C**). In addition, we found that CAR-mediated killing activity was associated with the elevated levels of IFN-γ production, as measured via ELISA, in the supernatant of HepG2 cells co-cultured with NK92MI/HN3 cells (**Fig 3D**). To further validate the cytotoxicity of NK92MI/HN3 cells, we co-cultured the NK92MI/HN3 cells with HepG2 cells by utilizing different effector-to-target (E:T) cell ratios ranging from 1:1 to 10:1. As shown in **Fig 3E**, the NK92MI/HN3 cells effectively eliminated the tumor cells at different ratios *in vitro*. To explore the *in vivo* anti-tumor activity of NK92MI/HN3 and NK92MI/HS20 cells, we established a xenograft model using immunodeficient (NOD/SCID) mice bearing subcutaneous HepG2 cells. Approximately 2 weeks after tumor cell inoculation, mice were grouped and treated with PBS, NK92MI, NK92MI/HN3 or NK92MI/HS20 cells. The results showed that administration of NK92MI/HN3 effectively inhibited the growth of the HepG2 xenografts (**Fig 3F**), indicating the effective anti-tumor activity of NK92MI/HN3 cells.

### 3.4 Impact of irradiation on anti-tumor efficacy of NK92MI/HN3 *In Vitro* and *in vivo*

**Fig 4:**
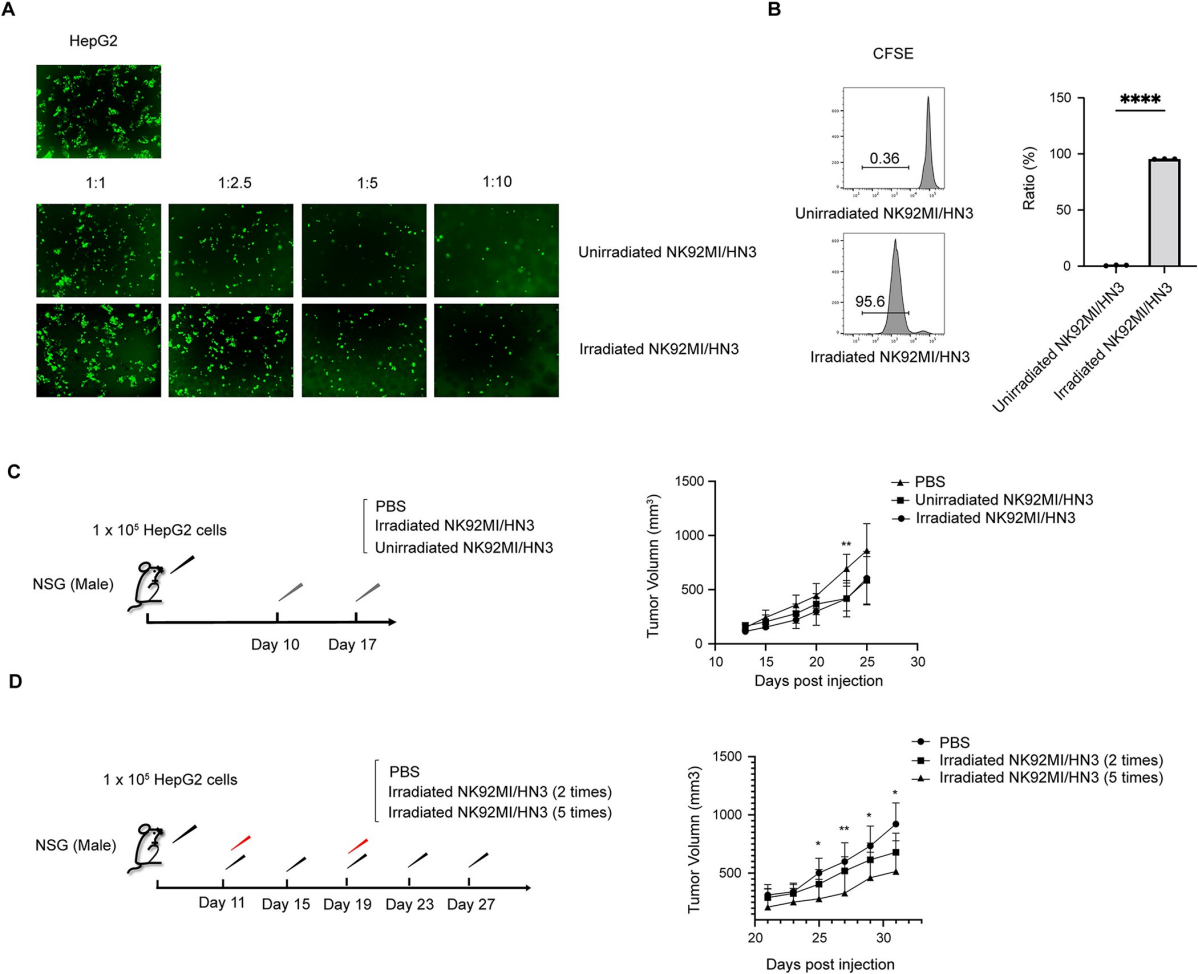
Impact of irradiation on anti-tumor efficacy of NK92MI/HN3 *in vitro* and *in vivo*. (A) Cytotoxicity activity of irradiated and unirradiated NK92MI/HN3 cells against HepG2 cells at an E:T ratio of 1:1, 2.5:1, 5:1 and 10:1. (B) CFSE-based proliferation assay of irradiated and unirradiated NK92MI/HN3 cells. (C) Procedure and growth curve of HepG2 Xenografts treated with PBS, irradiated NK92MI/HN3 or unirradiated NJ92MI/HN3 cells. (D) Treatment procedure optimizing of irradiated NK92MI/HN3 cells. n = 5 mice per group. Data are represented as mean ± SD. *, P < 0.05; **, P< 0.01; ***, P< 0.001.

To ensure the safety and minimize the risk of NK lymphoma development in patients, NK92 cells are commonly irradiated prior to clinical application. Studies have shown that a radiation dose of 5Gy is sufficient to limit the lifespan of CAR-NK92 cells while maintaining their effectiveness [44]. Accordingly, we assessed the specific cytotoxicity of both irradiated and unirradiated CAR-NK against HepG2 cells *in vitro* and *in vivo*. It was observed that both irradiated and non-irradiated NK92MI/HN3 cells displayed cytotoxicity effects against HepG2 cells at varying effector-to-target ratios *in vitro* (**Fig 4A**). To evaluate the impact of 5Gy irradiation on the longevity and proliferation of NK92MI/HN3, we performed a CFSE-based proliferation assay. Flow cytometry analysis showed that while irradiated NK92MI/HN3 cells remained viable for 5 days post-irradiation, they were unable to proliferate (**Fig 4B**). Furthermore, both irradiated and non-irradiated NK92MI/HN3 cells effectively inhibited tumor growth in HepG2 xenograft models (**Fig 4C**). These results suggest that irradiation does not compromise the anti-tumor efficacy of NK92MI/HN3 cells, as the irradiated NK92MI/HN3 cells displayed similar anti-GPC3 malignancy activity to unirradiated NK92MI/HN3 cells. To further optimize the treatment procedure, we administered irradiated NK92MI/HN3 cells to HepG2 xenografts every 4 days, for a total five-time injections. It was observed that this treatment regimen significantly inhibited the tumor growth (**Fig 4D**). Thus, the use of irradiated NK92MI/HN3 cells as a safe and effective option for CAR-NK immunotherapy in the treatment of HCC, preserving both safety and therapeutic efficacy.

### 3.5 Identification and analysis of GPC3 isoforms in HCC

**Fig 5:**
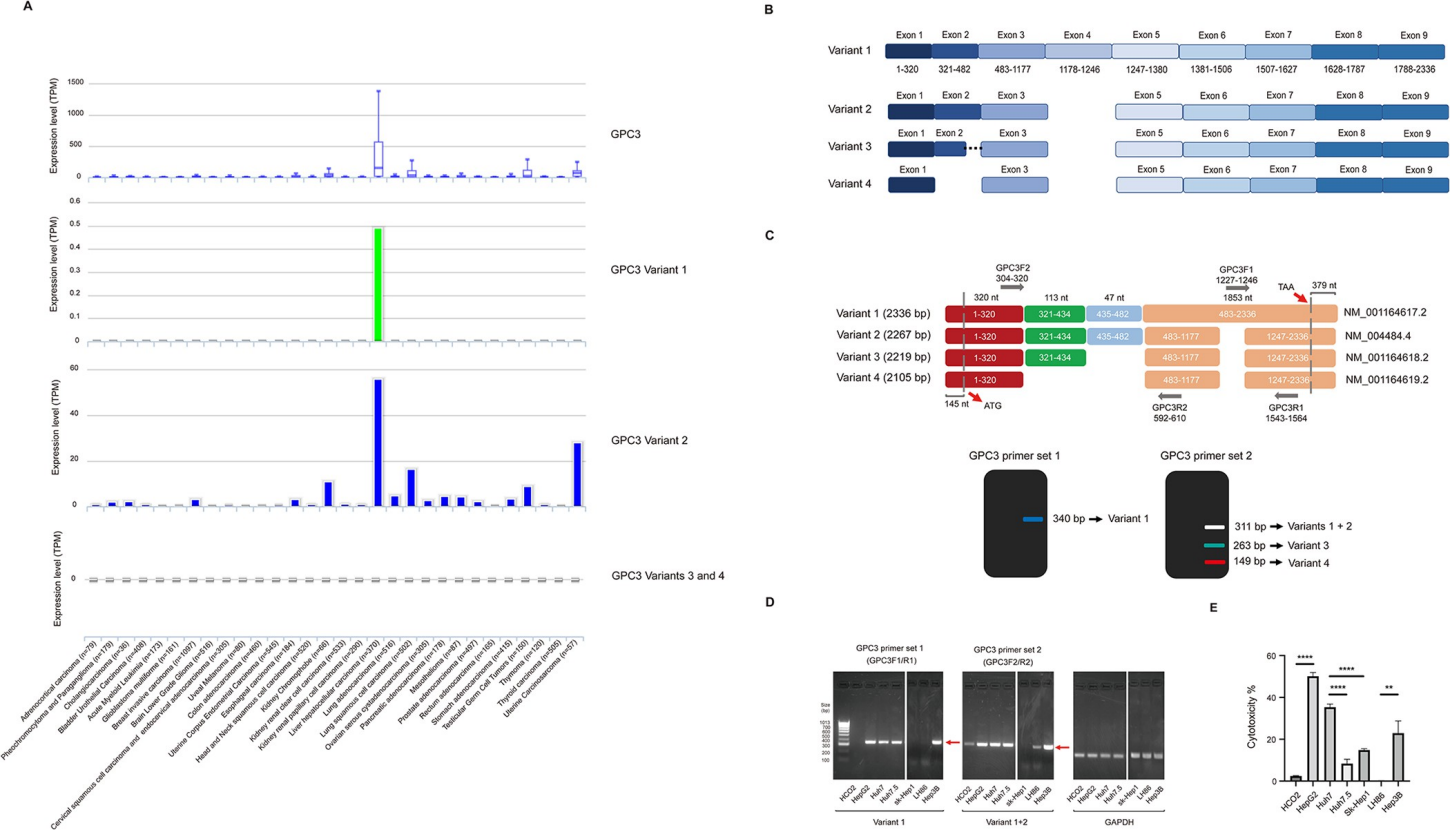
Identification and analysis of GPC3 isoforms in HCC. (A) Analysis of GPC3 and its variants in cancer. A web-based platform, ISOexpresso, for isoform-level expression analysis in human malignancies. (B) Sequence comparison between different GPC3 variants. (C) Designing specific primers for recognizing specific GPC3 variants. Band sizes of the PCR products representing different GPC3 variants are shown in the graph, using GPC3 primer sets 1 and 2. (D) Distribution and composition of GPC3 variants in different HCC cell lines. (E) Different killing activities of NK92MI/HN3 cells to various HCC cells. The mRNA levels were normalized with the mRNA levels of GAPDH. Data are represented as mean ± SD. *, P < 0.05; **, P< 0.01; ***, P< 0.001.

To analyze the expression of GPC3 and its variants in malignancies, we performed expression analysis with ISOexpresso, a web-based platform for isoform-level expression analysis in cancer [45]. The results showed that the mRNA level of GPC3 was markedly increased in HCC and is also detectable in several other cancers, suggesting a broad-spectrum activity of GPC3 in tumor development. In addition, the subtype expression profile data showed that GPC3 variants 1 and 2 are present in a variety of human malignancies, while GPC3 variants 3 and 4 are not (**Fig 5A**). This discrepancy presence may contribute to the differential regulation of GPC3 variants during tumor growth. Moreover, unlike GPC3 variant 2, variant 1 was only found in HCC, implying that GPC3 variant 1 presence may exert distinct biological activities in the development of HCC. In contrast, the expression level of GPC3 variant 2 showed much higher than that of GPC3 variant 1 in HCC, which is considered to be the dominant form of existence [29].

It was reported that there exist different kinds of GPC3 variants in HCC (**Fig 5A**), whereas no obvious evidence to show the impact of different GPC3 isoforms on cytotoxicity of CAR-NK cells. To explore the potential impact of GPC3 isoforms on anti-tumor efficacies, we first compared the sequence information of four GPC3 variants (NM_001164617.2, NM_004484.4, NM_001164618.2, and NM_001164619.2), which have been identified in GenBank and encode alternatively spliced forms. Through the sequence comparison, it was found that the main difference among the GPC3 isoforms was the deletion of part or entire sequence of exon 2 and exon 4 (**Fig 5B**). This exon loss phenomena could potentially result in different biological functions of GPC3 in the development of HCC. Based on sequence information, we designed two primer sets, GPC3F1/R1 and GPC3F2/R2, to examine the existence and distribution of GPC3 variants in HCC cell lines. The GPC3 variant 1 was detected with the GPC3F1 and GPC3R1 primer set at the size of 348 bp. The total GPC3 variants 1 and 2, variant 3, and variant 4 were detected at the size of 311bp, 286bp and 145bp, respectively, using the GPC3F2 and GPC3R2 primer set (**Fig 5C**). The results showed that GPC3 variants 1 and 2 were detected in HepG2, Huh7, Huh7.5, and Hep3B cells, only GPC3 variant 2, but not variant 1, was present in HCO2 and LH86 cells (**Fig 5D**). There were no GPC3 variant expression detected in Sk-Hep1 cells. Interestingly, we found that NK92MI/HN3 cell showed different killing activities to different HCC cells (**Fig 5E**), indicating that GPC3 isoform existence may have potential impact on anti-tumor efficiencies.

### 3.6 Impact of GPC3 isoforms on the cytotoxicity activity of NK92MI/HN3 cells

**Fig 6:**
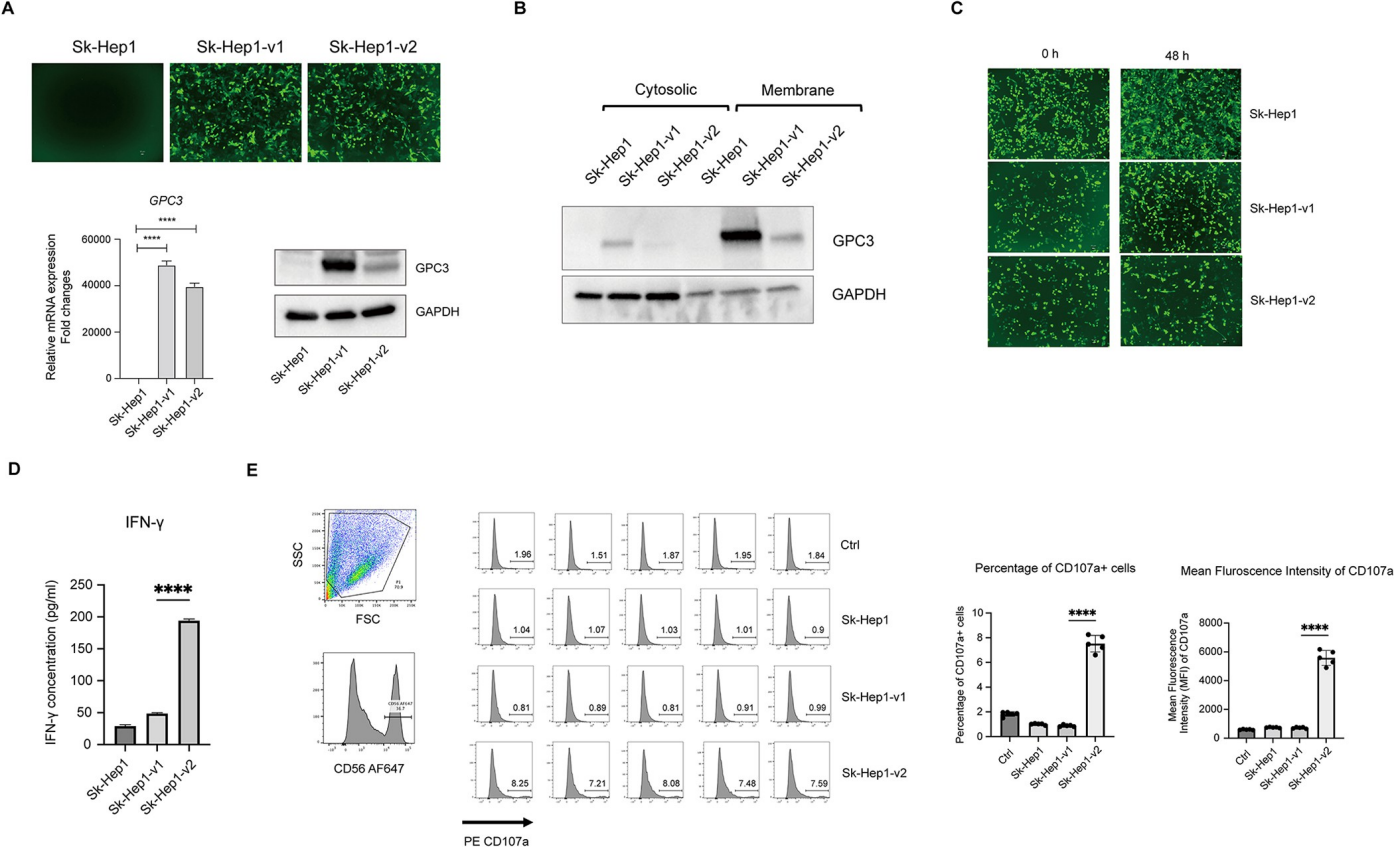
Cytotoxicity activity of NK92MI/HN3 cells to Sk-Hep1-v1 and Sk-Hep1-v2 cells. (A) Establishment of GPC3 variant overexpression cell model. Construction of the lentiviral vector containing GPC3 variant 1 and variant 2, respectively. The cells were checked for fluorescence signal under a microscope. The expression validation of GPC3 expression levels. (B) The assessment of GPC3 expression on the membrane and in the cytosolic of Sk-Hep1, Sk-Hep1-v1 and Sk-Hep1-v2 cells. (C) NK92MI/HN3 cells were co-cultured with Sk-Hep1, Sk-Hep1-v1 or Sk-Hep1-v2 cells at a E:T ratio of 5:1 for 12h, 24h and 48h. Cytotoxicity efficacies of NK92MI/HN3 cells were observed under the florescence microscope. (D) IFN-γ production was measured via ELISA in the supernatant. (E) CD107a expression levels were assessed by flow cytometry. The mRNA levels were normalized with the mRNA levels of *GAPDH*. Data are represented as mean ± SD. *, P < 0.05; **, P< 0.01; ***, P< 0.001.

Since GPC3 isoforms are not detectable in Sk-Hep1 cells, it would be a good model to generate two stable cell line Sk-Hep1-v1 and Sk-Hep1-v2 that were overexpressing with GPC3 variants 1 and GPC3 variant 2, respectively, to elucidate different biological activities on regulating HCC tumorigenesis and potential anti-tumor efficacies of CAR-NK92MI cells. To assess the expression levels of GPC3, we collected both protein and mRNA from Sk-Hep1, Sk-Hep1-v1, and Sk-Hep1-v2 cells for western blot and qPCR, respectively. Notably, we found that the GPC3 protein expression level of Sk-Hep1-v1 cells was significantly higher than in Sk-Hep1-v2 cells, despite mRNA levels were comparable in both cell lines (**Fig 6A)**. To further investigate the cellular localization of GPC3 expression, its distribution on the cell membrane and in the cytosol was assessed. The results revealed that GPC3 variant 1 exhibited a higher level of expression on the cell membrane compared to GPC3 variant 2 (**Fig 6B**). These findings suggest that the existence of different GPC3 isoforms may impact the properties of the protein and cellular activities and could potentially influence the CAR-NK therapy efficacy.

To evaluate the killing efficacy of NK92MI/HN3 cells in relation to different GPC3 isoforms, we co-cultured NK92MI/HN3 with Sk-Hep1, Sk-Hep1-v1 and Sk-Hep1-v2 cells *in vitro* at an E/T ratio of 5:1. Fluorescence signal of the HCC cells was observed under the microscope, which was served as an indicator to evaluate the killing efficacy of NK92MI/HN3 cells. It was observed that NK92MI/HN3 cells exhibit high cytotoxic effects against Sk-Hep1-v2 than Sk-Hep1 and SkHep1-v1 cells (**Fig 6C**), followed by an increased IFN-γ production in the supernatant (**Fig 6D**), indicating that the presence of different isoforms may have an impact on therapy outcomes. To further validate the activity of NK92MI/HN3 cells, the expression of CD107a, a sensitive marker for NK cell functional activity, was analyzed by flow cytometry. Co-culturing NK92MI/HN3 cells with Sk-Hep1-v2 cells led to a significant upregulation of CD107a expression in NK92MI/HN3 cells (**Fig 6E**), indicating the enhanced NK cell cytotoxicity against Sk-Hep1-v2 cells, but not against Sk-Hep1-v1 cells. Thus, further analysis and investigation may be required to better understand the implications of GPC3 isoform 1 in the clinical outcome of HCC treatment with NK92MI/HN3 cells.

## 4. Discussion

HCC is a highly aggressive form of cancer, and curative treatment options are typically limited to patients. Therefore, novel strategies for the patients with HCC are urgently needed. One promising approach involves the use of chimeric antigen receptor (CAR) technology to harness cell-based immunity. In the study, we constructed two CAR-NK cell lines, NK92MI/HN3 and NK92MI/HS20 cells, using GPC3-targeting HN3 single-domain antibody or HS20 single-chain antibody fragment, respectively. Compared to NK92MI/HS20 cells, NK92MI/HN3 cells exhibited a higher cytotoxicity against HepG2 cells (**Fig 3**). This discrepancy in cytotoxicity may be due to differences in antibody recognition. The HN3 antibody targets a conformational epitope on the GPC3 core protein, which requires both the amino and carboxy terminal domains [37]. In contrast, the HS20 antibody recognizes the heparan sulfate chains on GPC3 [36]. As a result, HN3 is more potent because it binds directly to the GPC3 core protein, contributing to its enhanced cytotoxicity. However, despite the higher cytotoxicity observed *in vitro*, the *in vivo* tumor growth inhibition by NK92MI/HN3 cells was less significant. This discrepancy may be attributed to challenges in persistence and trafficking of NK92MI/HN3 cells to tumor sites, both of which are crucial for the *in vivo* efficacy of CAR-NK cell therapies. Additionally, the complex tumor microenvironment (TME) *in vivo*, which is absent in *in vitro* conditions, could play a role in modulating NK cell activity. Immunosuppressive factors and cell-cell interactions in the TME may diminish the efficacy of NK92MI/HN3 cells *in vivo*, reducing their ability to effectively target and kill tumor cells. To overcome these challenges, further optimization of the dosing schedule and treatment regimen may be necessary to enhance the persistence and trafficking of CAR-NK cells. Potential strategies include repeated dosing, combining CAR-NK therapy with TME-modulating agents, or implementing genetic modifications to enhance CAR-NK cell survival and activity, which could improve the therapeutic outcomes in HCC.

Chimeric antigen receptor (CAR) T cell therapy is recognized as a promising immunotherapeutic strategy, especially in the treatment of relapsed and refractory B-cell malignancies [46]. However, CAR-T cell therapy faces several challenges. One of the major obstacle is the need to collect and utilize autologous cells, which requires labor-intensive processes, including isolation and *in vitro* expansion [47,48]. This concern may limit the broader clinical application of CAR-T-cell therapy, leading to increased interest in exploring alternative CAR platforms [49]. The modification of NK cells with CARs is being investigated as an alternative to T cells in various therapeutic areas. One advantage of NK cells over T cells is their non-major histocompatibility complex (MHC) restriction, which enables them to activate or inhibit target cells through germline-encoded activating or inhibitory receptors that interact with specific ligands on the target cells [50]. This characteristic makes NK cells suitable as an “off-the-shelf” product for patients in a cost-effective manner, as allogenic NK cells have shown a reduced risk of alloreactivity. Allogeneic NK cells can contribute to a graft-versus-tumor effect without causing GvHD, as shown in mouse models [51] and clinical studies [52]. Various sources of NK cells are being used to generate CAR-NK cells, including primary NK cells from cord blood, iPSC-derived NK cells, and NK cell lines [44]. In this study, we utilized the NK92MI/HN3 cell line to develop NK92MI/HN3 cells. NK92MI cells demonstrated sustained activity and proliferation rates after flow cytometry sorting or recovery from liquid nitrogen cryopreservation. As a result, the NK92MI cells are more amenable to stable in vitro expansion and exhibit higher CAR transduction efficiency, making them a convenient source of NK cells for the production of CAR-NK cells. However, it is important to note that NK92MI cells, a modified IL-2-independent NK92 cell line derived from an NK lymphoma patient, is indeed a human cancer cell line. This presents potential risks related to uncontrolled growth and the possible formation of secondary malignancies. Although NK92MI cells have shown promise in clinical studies, their safety in clinical settings requires careful evaluation and further assessments. To mitigate the risk of secondary NK lymphoma development in patients, large doses of radiation are applied to NK92MI cells prior to clinical use. While this approach ensures safety, it also reduces the cytotoxicity of NK92 cells. Studies have identified a radiation dose of five Gy as optimal for balancing the safety and effectiveness of the CAR-NK92MI cells [44].

GPC3 plays a crucial role in HCC carcinogenesis, and human GPC3 gene can be alternatively spliced into four variants, however, the biological significance of these variants in HCC and therapies remains largely unknown. In the study, various HCC cell lines were analyzed for the distribution of GPC3 variants. Consistent with the database, GPC3 variant 2 was found in a broader range of cells, suggesting it is more commonly expressed [29]. Studies have demonstrated that alternative splicing generates multiple protein isoforms with distinct biological properties, such as protein interaction, subcellular localization, and catalytic ability [31]. These differences in isoform expression can lead to alterations in cellular behavior, influencing processes like cell proliferation and therapy responsiveness [32]. Several studies have demonstrated that the abnormal regulation of alternative splicing contributes to tumor occurrence and development in human malignancies [53–56]. In addition to the role of alternative splicing in tumorigenesis, that alternative splicing variants are implicated in resistance to immunotherapy [30,55,57]. In the study, we observed that NK92MI/HN3 exhibited varying cytotoxic efficacies against Sk-Hep1-v1 and Sk-Hep1-v2 cells (**Fig 6**). This was accompanied by increased IFN-γ production and CD107a expression. These discrepancies suggest that the existence of GPC3 alternative splicing variants may increase isoform diversity, potentially impacting therapy outcomes. Understanding the diversity of GPC3 isoforms and their influence on treatment efficacy is critical. The differential cytotoxicity observed between HCC cells expressing GPC3 isoform 1 and isoform 2 underscores the importance of considering GPC3 isoform expression patterns in patient populations. Validating GPC3 isoform profiles could help understand the potential mechanism of escape and improve the precision of CAR-NK therapy, ensuring both efficacy and safety in clinical trials. This approach could optimize therapeutic outcomes and enhance the overall effectiveness of CAR-NK treatments for HCC. Additionally, combining CAR-NK therapy with other treatments, such as immune checkpoint therapy, may improve NK cell antitumor activity and enhance long-term complete remission in these patients.

In this study, we demonstrated the anti-tumor effects of GPC3-specific NK92MI/HN3 cells both *in vitro* and *in vivo*. Our findings emphasize the therapeutic potential of targeting GPC3 in HCC using CAR-NK cells, particularly in light of the observed variation in cytotoxic efficacy between different GPC3 isoforms. These results suggest that isoform profiling may be crucial in optimizing CAR-NK therapies for better patient outcomes. Further exploration of GPC3 isoform diversity in clinical settings and its impact on therapy resistance could guide the development of more personalized and effective immunotherapies for HCC.

## Supporting information

S1 Raw images (PDF)
