# Supplementary material for: Exploring Glypican-3 targeted CAR-NK treatment and potential therapy resistance in hepatocellular carcinoma
Source: PLoS One. 2025 Jan 22;20(1):e0317401. doi: 10.1371/journal.pone.0317401 (PMC11753693; doi:10.1371/journal.pone.0317401)

Figure 3B

Sample: HCO2, HepG2, Huh7, Huh7.5, Sk-Hep1, LH86 and Hep3B

GAPDH

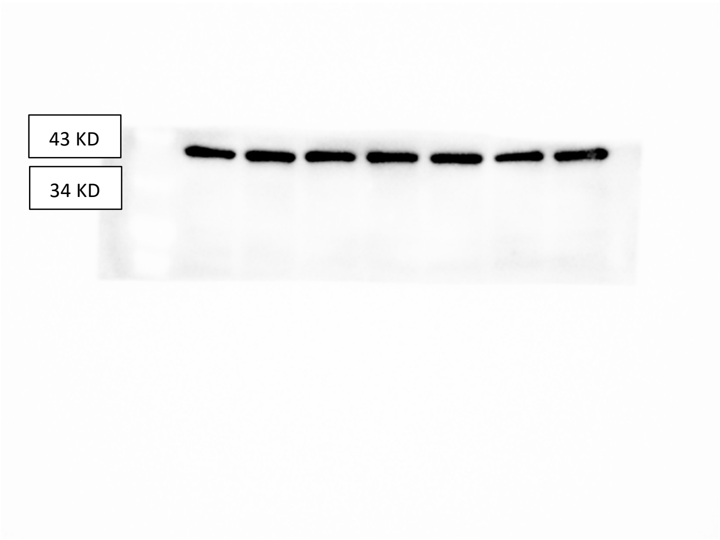

GPC3

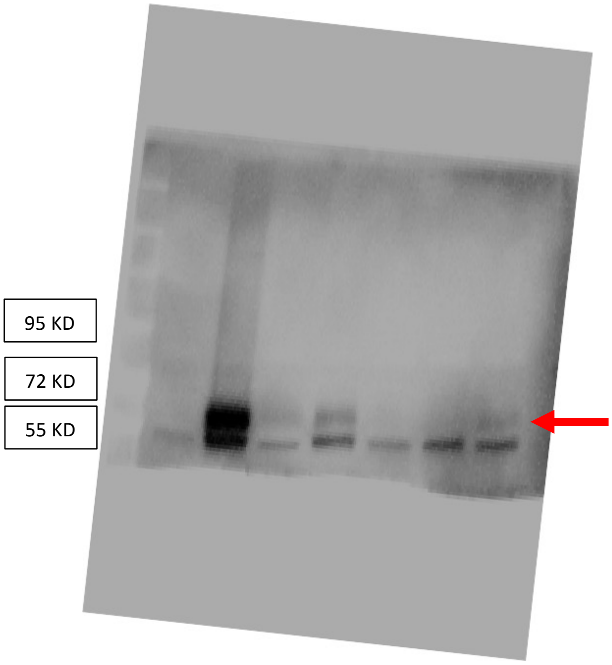

Figure 5D

HCO2, HepG2, Huh7, Huh7.5

GPC3 primer set 1/ GPC3 primer set 2/GAPDH

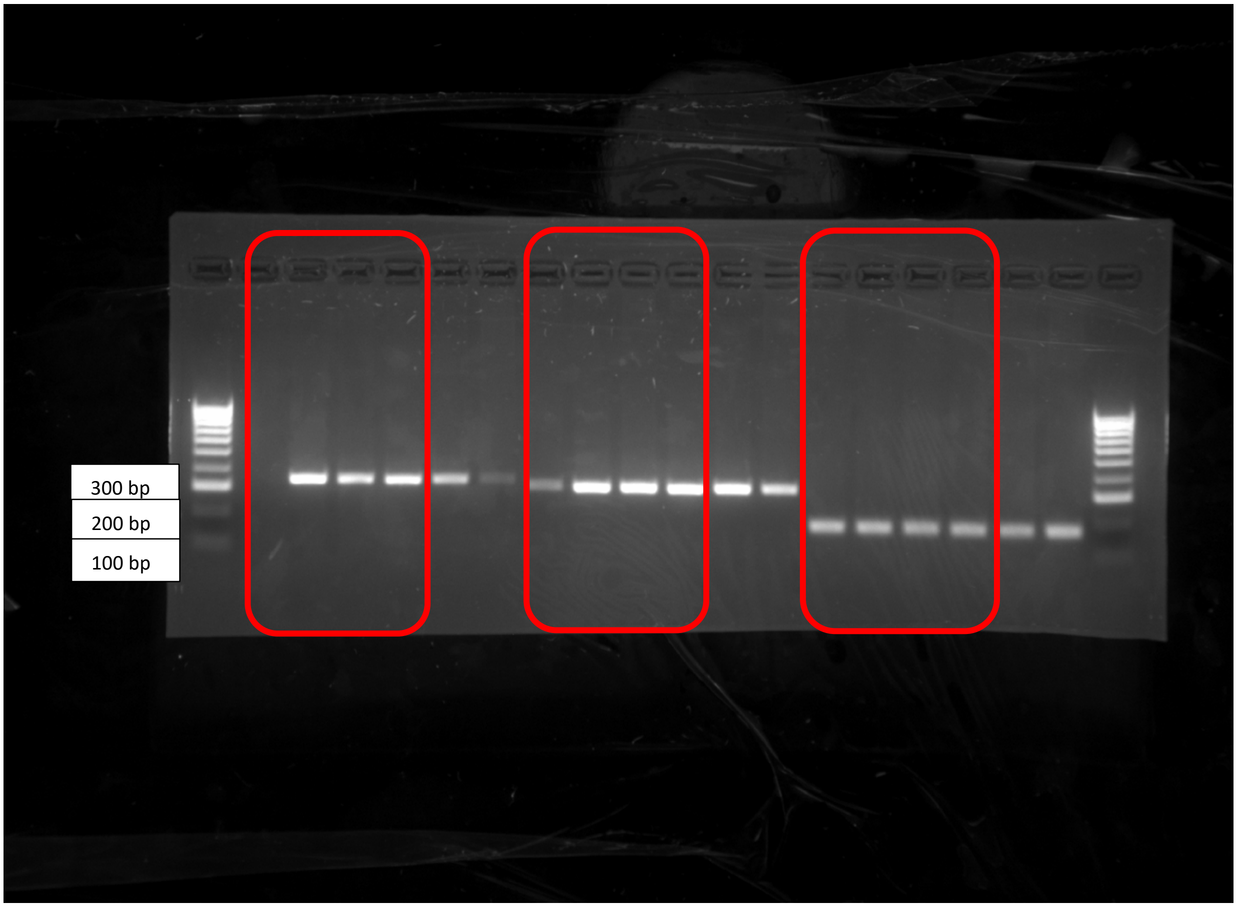

Figure 5D

Sk-Hep1, LH86, Hep3B

GPC3 primer set 1/ GPC3 primer set 2/GAPDH

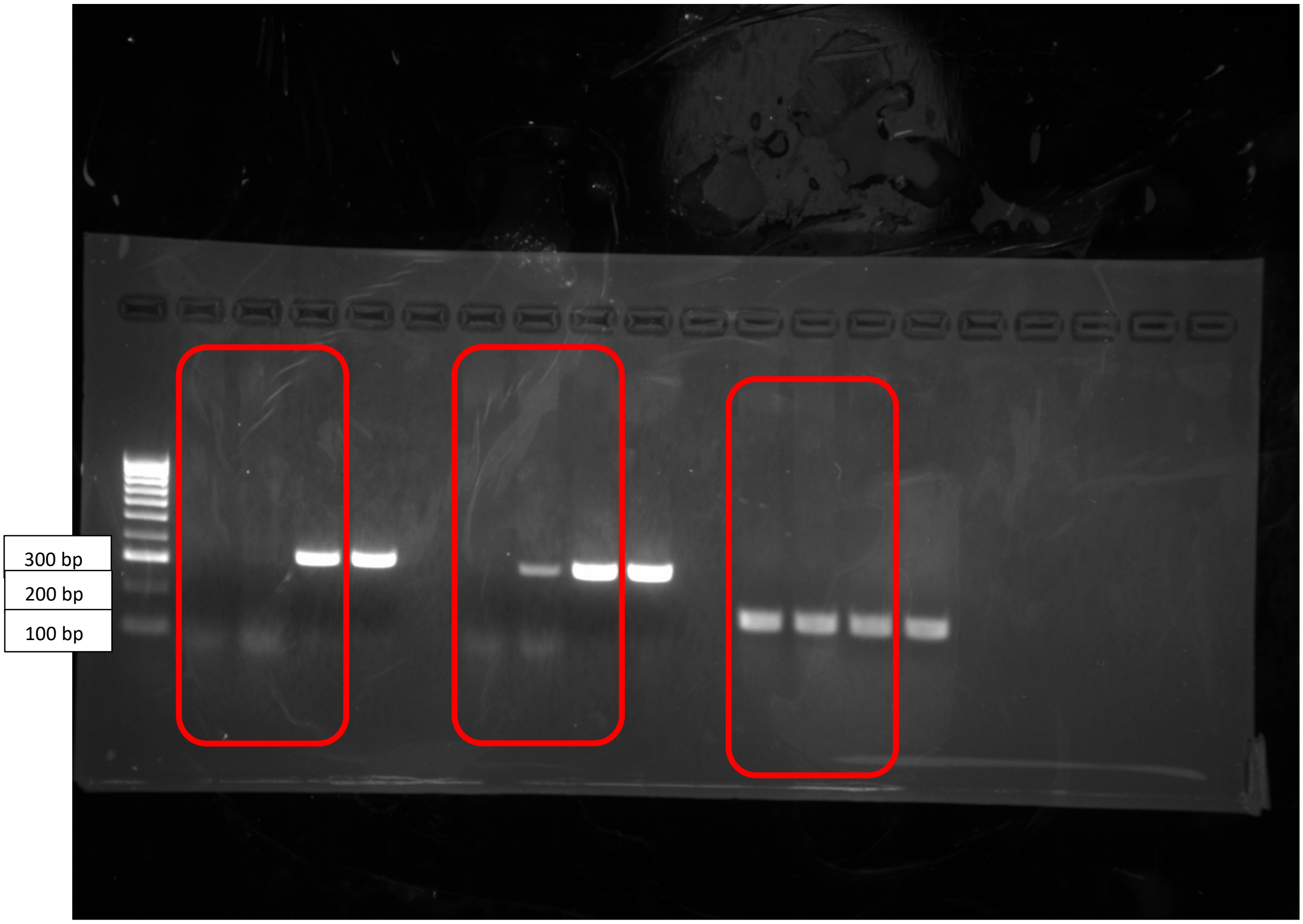

Figure 6A

Sk-Hep1, Sk-Hep1-v1 and Sk-Hep1-v2

GAPDH

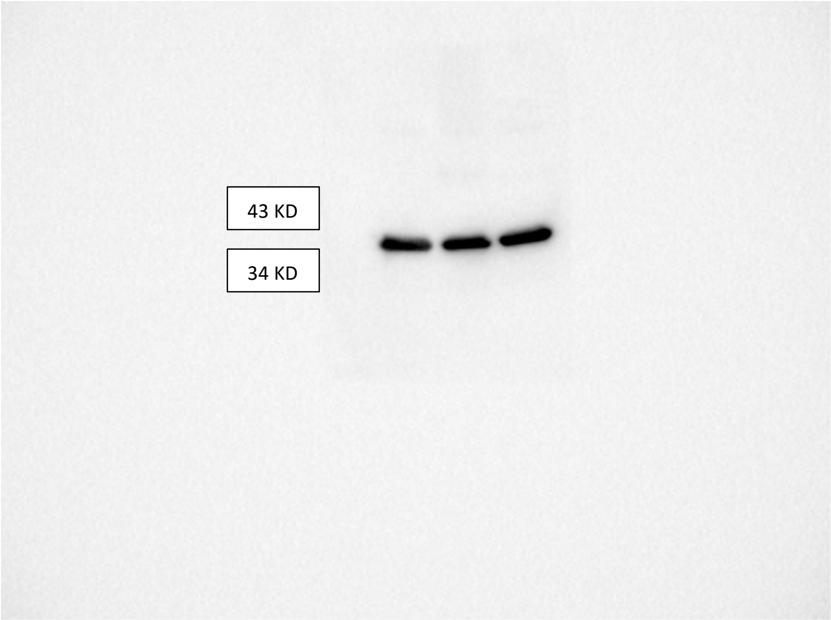

GPC3

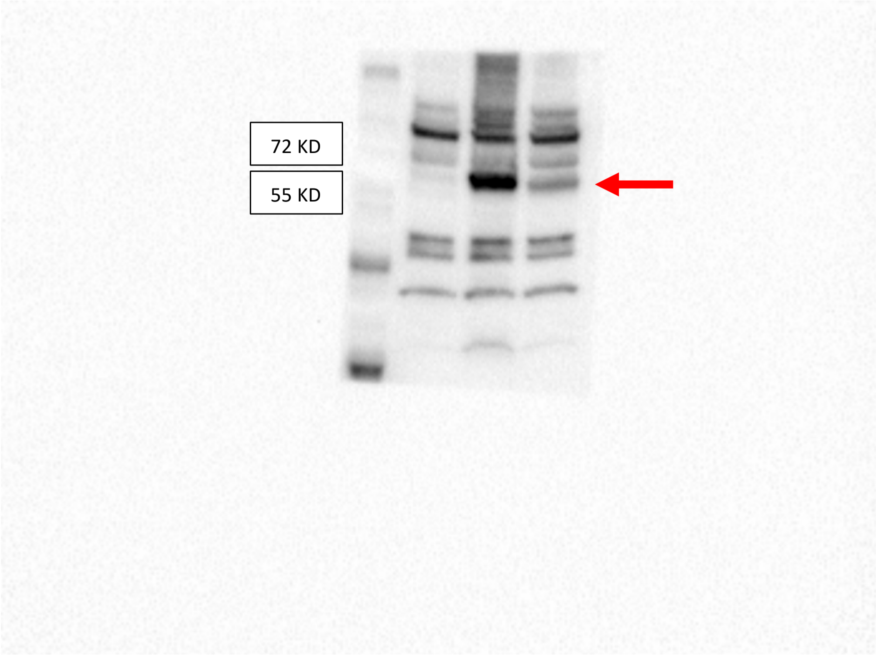

Figure 6B

Cytosolic (Sk-Hep1, Sk-Hep1-v1 and Sk-Hep1-v2) and Membrane (Sk-Hep1, Sk-Hep1-v1 and Sk-Hep1-v2)

GAPDH

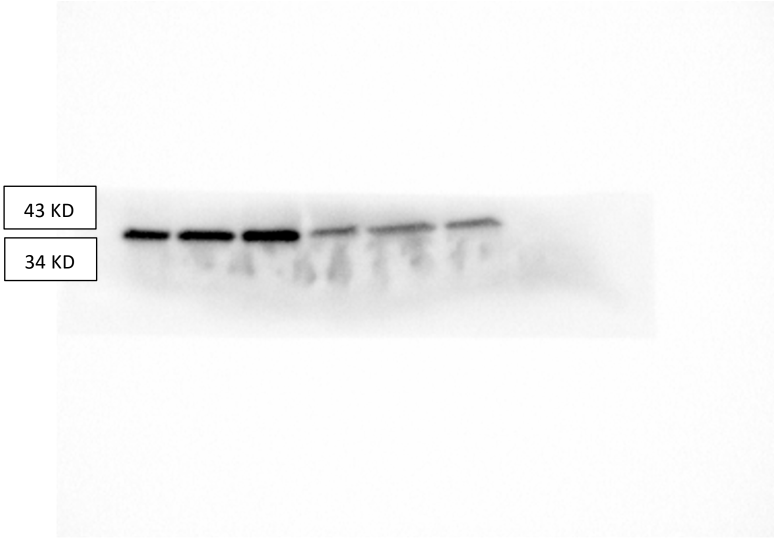

GPC3

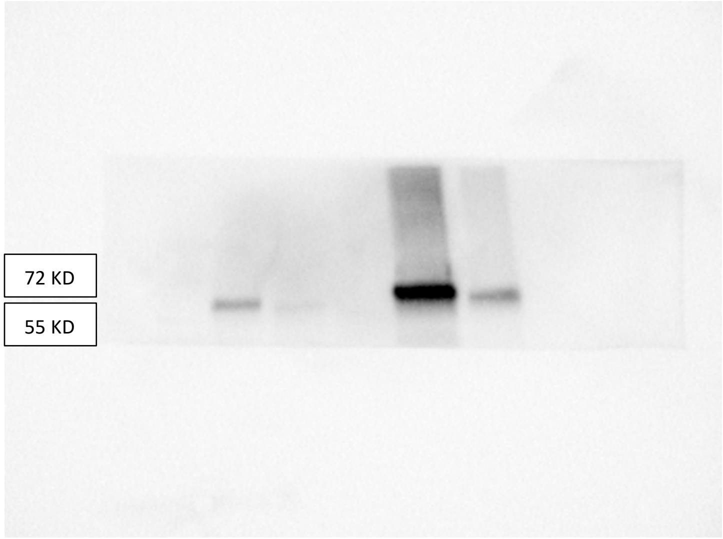

Supplement: S1 Raw images — (PDF) [file pone.0317401.s001.pdf]
